# Novel Electrotrichogenic Device Promotes Hair Growth in Men With Androgenetic Alopecia: A Pilot Study

**DOI:** 10.1111/jocd.70202

**Published:** 2025-04-28

**Authors:** Samuel Jellard, Stephanie Moore, Carlos Andrés Chacón‐Martínez

**Affiliations:** ^1^ Mane Biotech GmbH Cologne Germany; ^2^ Surrey Trichology Clinic Surrey UK

**Keywords:** cosmetic dermatology, hair growth, male pattern baldness

## Abstract

**Background:**

Androgenetic alopecia (AGA) is the most common cause of hair loss globally, affecting millions of people, particularly men. Current treatments include FDA‐approved drugs and devices, but many patients experience side effects or suboptimal results. The niostem device is a new, wearable device that delivers low‐level electrical stimulation to promote hair growth. This pilot study aims to evaluate the efficacy and safety of the niostem device in male AGA patients.

**Methods:**

A total of 21 male patients with AGA used the niostem device daily for 30 min over 6 months. Participants had not used any anti‐hair loss products within the 6 months preceding the start of the study. Hair density, thickness, and terminal hair counts were assessed at baseline, 3 months, and 6 months using trichoscopic measurements. Patient‐reported outcomes were recorded, and adverse events were monitored.

**Results:**

The niostem device resulted in significant increases in hair count, with a 12% increase in total hair density at 3 months and a 19.3% increase at 6 months. Hair thickness also increased by 8.8% in 6 months. Terminal hair density improved significantly over time, with visible hair growth observed in the participants. No adverse events were reported.

**Conclusions:**

The niostem device demonstrated a significant increase in hair density and hair thickness in male AGA patients, with no adverse effects. Further large‐scale studies are warranted.

## 1 Introduction

Androgenetic alopecia (AGA), or male and female pattern baldness, is the most prevalent form of hair loss worldwide, affecting approximately 50% of men and 25% of women by the age of 50 [1]. The condition is characterized by the progressive miniaturization of hair follicles due to genetic sensitivity to dihydrotestosterone (DHT), the most potent form of testosterone [2]. This miniaturization causes a shift from terminal hairs (thick and pigmented) to vellus hairs (thin and short) over time, resulting in thinning hair and eventually baldness [3].

The human skin is a closely interlinked biological framework that consists of a network of appendages, such as the hair follicles, where hair growth takes place. This framework is constantly regenerated to heal and renew skin and hair. The key drivers behind this are adult stem cells known as hair follicle stem cells (HFSCs), which have inherent regeneration capacity [4, 5] and generate hair in cycles in successive phases of growth (anagen) and rest (telogen) [6]. Prolonged exposure to DHT and aging progressively slows HFSC activity across the scalp of individuals with AGA, which leads to hair follicle growth arrest, transformation of terminal hair into thinner hair known as vellus hair, increased hair shedding, and hair follicle shrinkage [7]. Interestingly, even bald scalps contain more than 100 000 miniaturized hair follicles that could be reactivated to achieve hair regrowth.

Current treatments for AGA include topical solutions like minoxidil, oral medications such as finasteride, and devices such as low‐level laser therapy (LLLT). While these treatments are FDA‐approved, they often have limited efficacy and can produce side effects such as sexual dysfunction with finasteride or scalp irritation with minoxidil [8, 9]. Hair transplantation remains an option but is invasive and expensive [10]. Thus, there is a growing need for non‐invasive, effective, and side‐effect‐free alternatives.

Electrotrichogenesis (ETG) is the use of low‐level electrical fields to stimulate hair growth. This technology has been shown to have positive hair regeneration effects on human dermal papilla cells in vitro [11], as well as to increase hair growth in rats [12] and mice [13]. Thus, electrical stimulation has emerged as a promising safe and effective treatment for hair loss, with studies in humans showing that it can promote hair regrowth in males with AGA and women with chemotherapy‐induced hair loss [14, 15, 16]. However, ETG has traditionally been confined to clinic‐based systems, limiting its accessibility and widespread adoption.

The niostem device is a novel device that uses low‐level electrical stimulation to stop hair loss and promote hair growth. Unlike other devices using electrical stimulation against hair loss, the niostem device is lightweight, portable, and designed for convenient at‐home use. It delivers the stimulation directly to the scalp, overcoming challenges associated with hair interference that often limit the effectiveness of clinic‐based systems and LLLT devices. In this pilot study, we aimed to evaluate the efficacy and safety of the niostem device for the treatment of AGA in men.

## 2 Materials and Methods

### 2.1 Study Design

Twenty‐two male participants aged 21–40 years with AGA (Norwood‐Hamilton scale II to VI) were recruited for this 6‐month open‐label study (Table 1). All participants were treatment‐naïve for at least 6 months before enrollment and agreed not to use any other hair loss treatments during the study. Two participants were excluded from the study: one due to a diagnosis of hypothyroidism, and one withdrew after 3 months but was included in the 0‐ and 3‐month analyses. The remaining 21 participants completed the study at the 3‐month mark, and 20 completed the full 6‐month study.

**TABLE 1:** Age and hair loss stage distribution of participants enrolled in this study.

Norwood Scale	Number of participants
2	5
3	0
3 vertex	4
4	6
5	3
6	4

Age (years)	Number of participants
21–30	10
31–40	12

Participants were instructed to use the niostem device for 30 min daily over a 6‐month period. Standardized global photography was performed at baseline (0 month), 3 months, and 6 months. Hair growth was assessed using trichoscopy and global photography. Participants' self‐assessments and adverse event reports were collected regularly.

The study was approved by the relevant institutional review board of the company (2019/PS024) and performed in compliance with the principles of the Declaration of Helsinki. All subjects participated voluntarily and provided written informed consent regarding the publication of photos and any information collected during the study.

### 2.2 The Niostem Device

**FIGURE 1:**
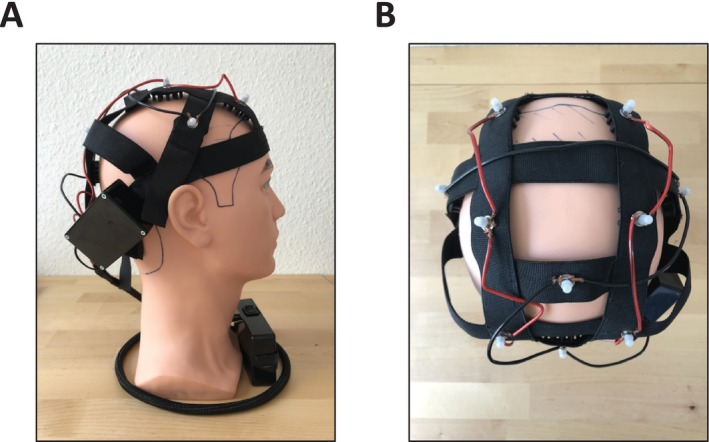
The niostem device. (A) Side and (B) upper view of the device.

Similar to other electrotrichogenic devices [16, 17], the niostem device delivers low‐level electrical stimulation to the scalp. This stimulation is delivered via conductive, brush electrodes that make direct contact with the scalp's surface (Figure 1A,B). The electrical stimulation is within the microampere range, ensuring high safety and comfort. The electrical pulses are short with a voltage between 1 and 160 V, a charge of between 1 nC and 1 μC within a pulse, and a frequency between 1 and 100 Hz. The device is designed for easy at‐home use, with 30‐min daily sessions for a period of 6 months. The niostem device is proprietary technology of Mane Biotech GmbH with the patent number EP4200009B1.

### 2.3 Hair Assessments

The primary endpoint of the study was the increase in hair density from baseline at 3 and 6 months. Secondary endpoints included changes in hair thickness and terminal hair density, as well as patient‐reported outcomes. Hair parameters were measured using trichoscopy, with hair counts performed in four adjacent 25 mm^2^ regions of interest on the scalp. Trichoscopic measurements were performed on areas with an intermediate degree of hair loss and were located from the nose bone along the vertex of the skull (Sagittal suture). A dot was marked with a permanent marker at its border to allow re‐assessment at exactly the same area, as described elsewhere [17]. The measurements were performed using a DinoLite camera and the DinoXcope software. Terminal hair density was determined by categorizing hairs into vellus (< 40 μm) and terminal (> 40 μm) hairs. Images were manually analyzed in a blinded manner by the independent, experienced trichologist (Surrey Trichology Clinic).

### 2.4 Statistical Analysis

For the subject that was excluded from the study, no data were used for the analyses. For a second subject that withdrew after the 3 month check, the recorded data were used for analyses. For comparisons between baseline, 3, and 6 months, a Mixed Effects Model with Tukey's multiple corrections test was used. *p* values are shown in the graph and legends as follows: ***p < 0.001, **p < 0.01, and *p < 0.05. Statistical analysis was conducted using GraphPad Prism 10 (GraphPad Software).

## 3 Results

### 3.1 Increased Hair Density and Thickness After 6 Months

**FIGURE 2:**
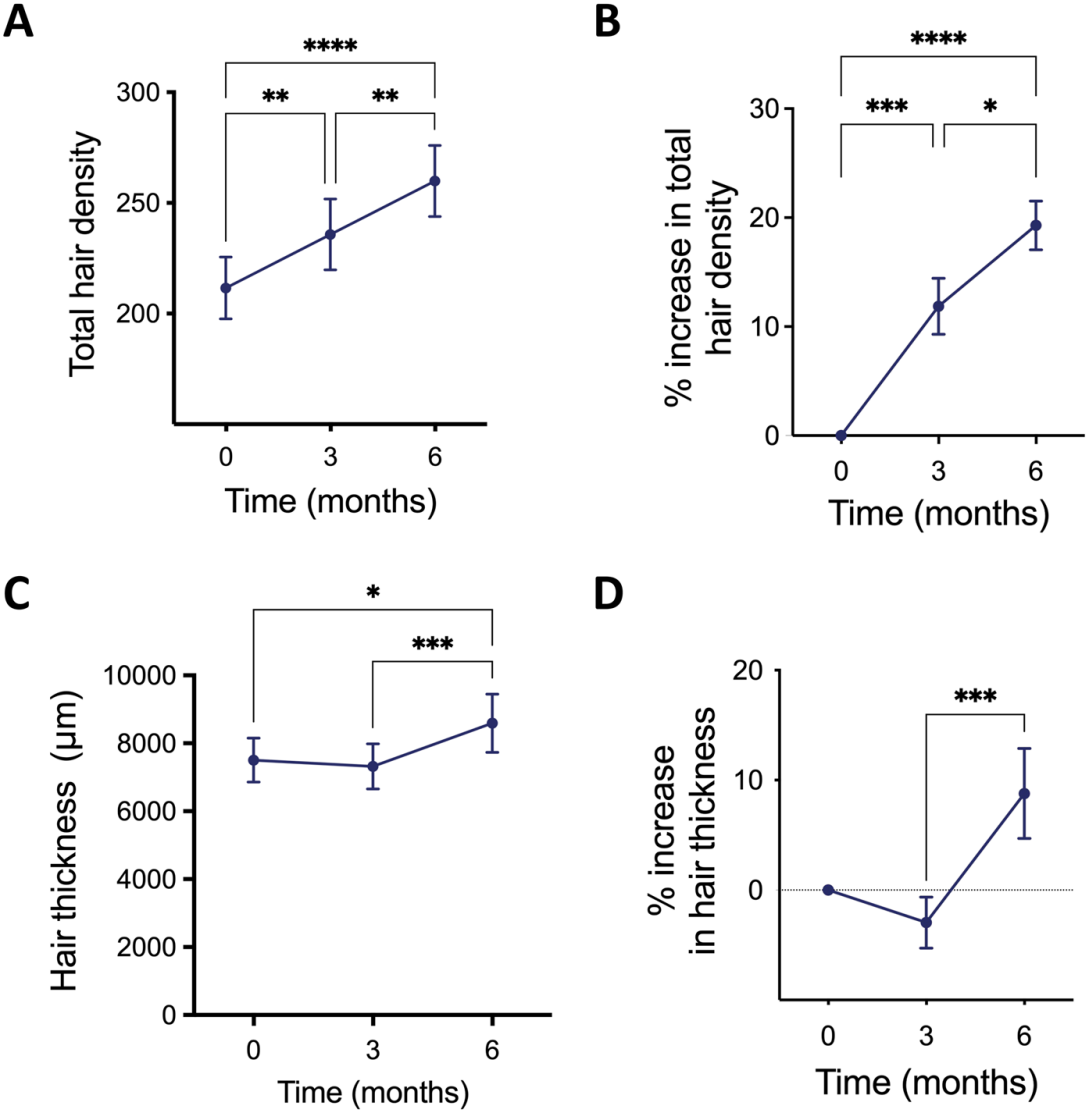
The niostem device increased hair density and thickness by 6 months. (A) Total hair counts increased by the niostem device by 3 and 6 months (Mixed Effects Model + Tukey's multiple corrections test; *n* = 20–21 testers; ***p < 0.001 and **p < 0.01). (B) Percentage increase in hair density compared to baseline at Month 0. The niostem device increased total hair counts by 12% and 19.2% at 3 and 6 months, respectively (Mixed Effects Model + Tukey's multiple corrections test; *n* = 20–21 testers; ***p < 0.001, **p < 0.01 and *p < 0.05). (C) Hair thickness increased upon use of the niostem device for 3 and 6 months (Mixed Effects Model + Tukey's multiple corrections test; *n* = 20–21 testers; ***p < 0.001 and *p < 0.05). (D) Percentage increase in hair thickness compared to baseline at Month 0. The niostem device increased hair thickness by 8.8% at 6 months (Mixed Effects Model + Tukey's multiple corrections test; *n* = 20–21 testers; ***p < 0.001).

After 3 months, 95.4% of participants stopped their hair loss and 86.3% of them showed hair regrowth, seen as an increase in hair density over the baseline. The participants showed a significant increase in total hair density with a mean increase of 12% compared to baseline (Figure 2A,B). In absolute hairs/cm^2^, there was an increase from 211.6 ± 13.42 at baseline to 235.8 ± 15.32 after 3 months (Table 2 and Table S1). After 6 months, 100% of participants showed hair regrowth. The mean increase in total hair density was 19.3% compared to baseline (Figure 2A,B). In absolute hairs/cm^2^, there was an average net increase of 48.3 ± 22.6 hairs/cm^2^, from 211.6 ± 13.42 at baseline to 259.9 ± 16.0 after 6 months.

**TABLE 2:** Changes in hair thickness and density over time.

Parameters	Baseline	3 months	6 months
Total hair density (hairs/cm^2^)	211.6 ± 13.42	235.8 ± 15.32	259.9 ± 16.06
Terminal hair density (hairs/cm^2^)	85.2 ± 11.59	77.9 ± 11.34	98.3 ± 15.06
Vellus hair density (hairs/cm^2^)	126.0 ± 13.41	157.2 ± 16.31	153.5 ± 18.00
Cumulative hair thickness (μm)	7504 ± 646.5	7321 ± 662.1	8591 ± 856.4

*Note:* *n* = 21 at baseline; *n* = 21 at 3 months; *n* = 20 at 6 months. Mean ± SEM are shown.

**FIGURE 3:**
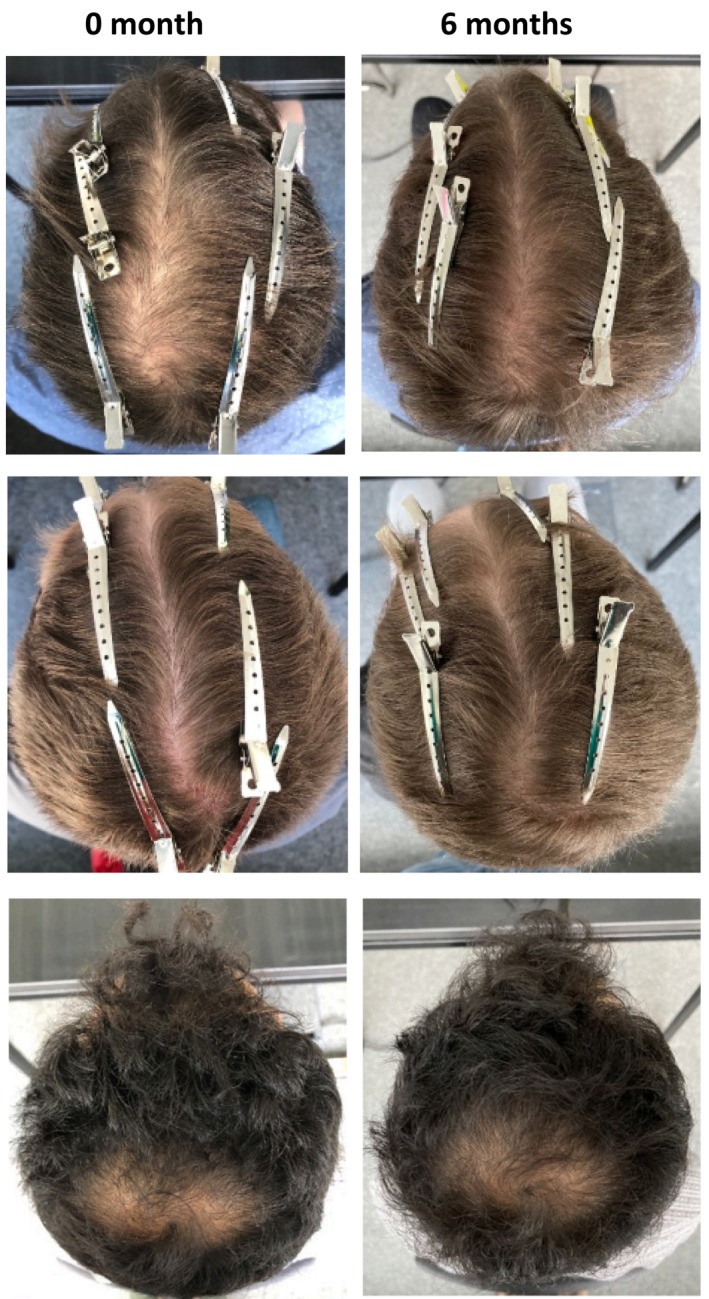
Representative images of testers at the beginning and end of treatment. (A) 31–40 years old, before and 6 months after. Change in total hair density +43.3%. (B) 31–40 years old, before and 6 months after. Change in total hair density+19%. (C) 21–30 years old, before and 6 months after. Change in total hair density +25.8%.

We next assessed whether hair thickness was also affected by the use of the device. While the cumulative hair thickness was statistically unchanged at 3 months (7504 ± 646.5 μm vs. 7321 ± 662.1 μm, baseline vs. 3 months, respectively), the cumulative hair thickness increased significantly from 7504 ± 646.5 μm at baseline to 8591 ± 856.4 μm by 6 months, which corresponds to an increase of 8.8% in hair thickness in 6 months (Figure 2C,D). Pictures of the participants' macroscopic hair improvements are shown in Figure 3 and Figure S1.

**FIGURE 4:**
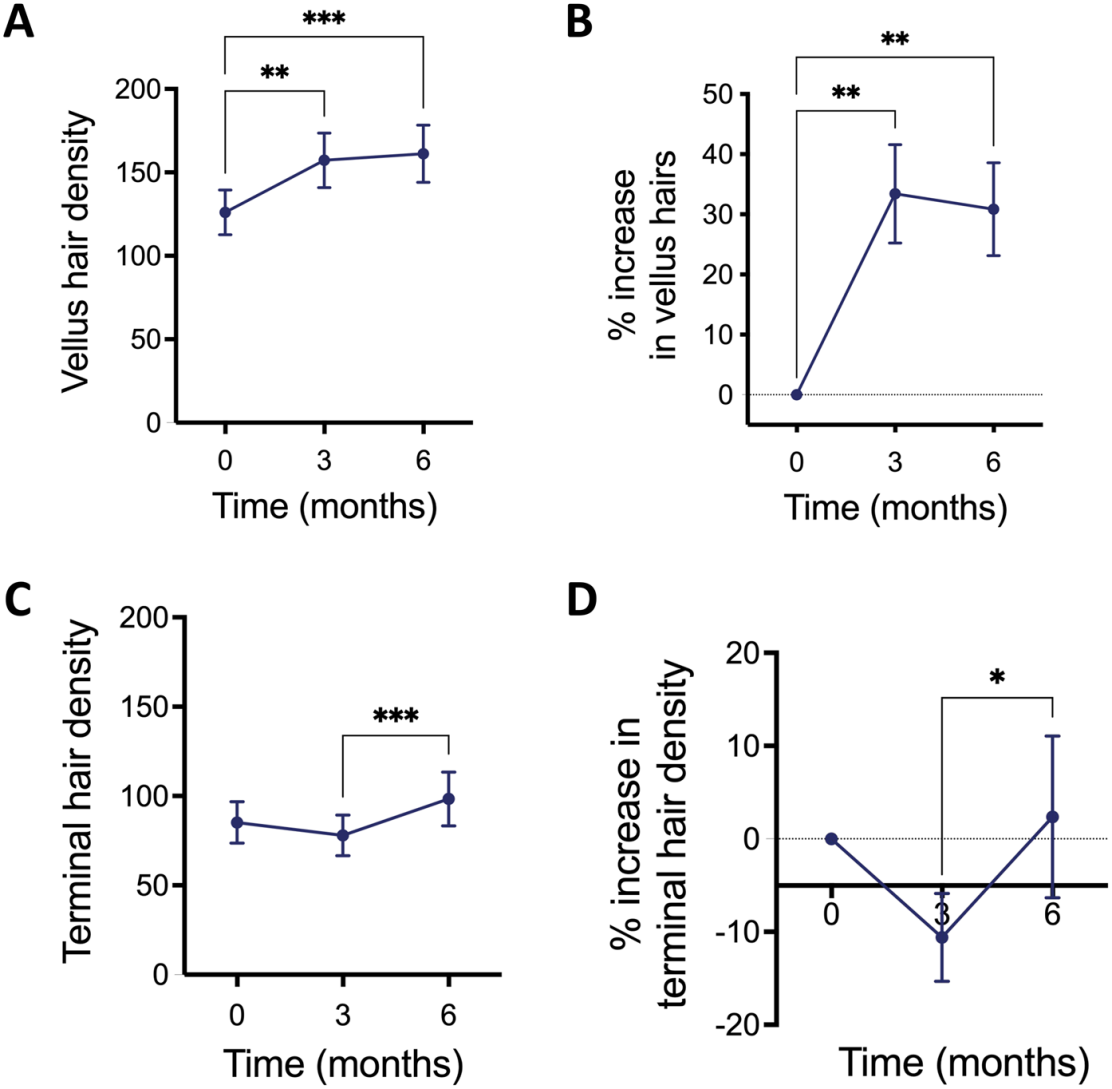
The niostem device increased vellus and terminal hairs at 3 and 6 months, respectively. (A) Vellus hair density was increased by the niostem device at 3 months, and no further changes were observed at 6 months (Mixed Effects Model + Tukey's multiple corrections test; *n* = 20–21 testers; ***p < 0.001 and **p < 0.01). (B) Percentage increase in vellus hair density compared to baseline at Month 0. The niostem device increased vellus counts by 33.4% and 30.84% at 3 and 6 months, respectively (Mixed Effects Model + Tukey's multiple corrections test; *n* = 20–21 testers; **p < 0.01). (C) Density of terminal hair was unchanged at 3 months, but increased at 6 months by the niostem device (Mixed Effects Model + Tukey's multiple corrections test; *n* = 20–21 testers; ***p < 0.001). (D) Percentage increase in the density of terminal hairs compared to baseline at Month 0. The niostem device did not significantly affect terminal hair density at 3 months, which was increased by 8.2% at 6 months (Mixed Effects Model + Tukey's multiple corrections test; *n* = 20–21 testers; *p < 0.05).

Since the increase in total hair density was concomitant with a slight, but not significant, decrease in hair thickness by 3 months, we quantified vellus (< 40 μm) and terminal (> 40 μm) hair density. After 3 months, there was a significant increase in the density of vellus hairs (Figure 4A,B), whereas the terminal hair density slightly decreased, but this change was not statistically significant (Figure 4C,D). Interestingly, at 6 months, the density of vellus hairs remained constant, whereas the density of terminal hairs significantly increased from 3 to 6 months (Figure 4C,D). These changes were accompanied by a significant increase in cumulative hair thickness by 6 months (Figure 1C,D).

### 3.2 Patient Self‐Perception

**FIGURE 5:**
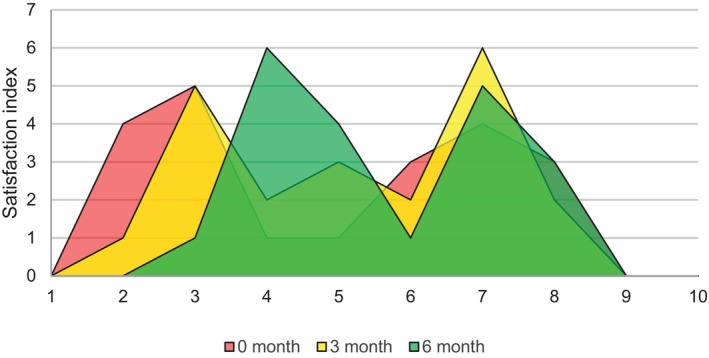
Participant's satisfaction index related to their hair condition. By the end of the study, the following question was asked: On a scale of 1–10, how satisfied are you with your current hair condition?

Patient self‐assessment scores improved significantly over time. At baseline, participants rated their satisfaction with their hair condition at an average of 4.9 on a 10‐point scale. By 3 months, the average score had increased to 5.6, and by 6 months, it reached 6.2 (Figure 5). Approximately 75% of participants reported feeling more confident and satisfied with their hair by the end of the study.

### 3.3 Adverse Events

Overall, the use of the device was well tolerated, and no serious side effects were reported. Minimal adverse effects included itchy scalp (scalp pruritus; 9%) and slight headache in the first 2–4 days after the initial use (4.5%), which are also common with approved anti‐hair loss drugs and LLLT devices [8]. Overall, the device demonstrated an excellent safety profile.

### 3.4 Compliance

Self‐reported device usage compliance was high and tended to decrease slightly after 3 months. In the first month, 86.4% of testers used the device 7×/week; by 3 months, 72.7% used it 7×/week, and by 6 months, 40% used 7×/week and 45% used it 6×/week. No difference in efficacy was observed among users with less frequent use of the device (7×/week vs. 6×/week).

## 4 Discussion

The results of this pilot study indicate that the niostem device is a promising new approach to combat male AGA, offering significant improvements in hair density and thickness with minimal side effects. Patient‐reported outcomes corroborated the objective hair assessments, with participants expressing increased satisfaction and confidence in their hair appearance over time. The ability to track progress using the companion app likely contributed to high adherence rates, with most participants using the device consistently throughout the study.

The results of this study corroborate previous results on the good efficacy and safety of ETG for promoting hair growth in men with AGA [15, 16]. Previous clinical studies in male AGA patients have demonstrated a 7%–14% increase in total hair density after 4–12 months of treatment with finasteride [18, 19], minoxidil [20, 21], and LLLT devices [22, 23]. In comparison, the present study showed a 19.2% increase in hair density and an 8.8% increase in hair thickness after 6 months of using the niostem device, showing that this approach holds considerable promise in combating AGA. Importantly, these improvements were achieved without the side effects commonly associated with pharmacological treatments. On the other hand, the study participants found the niostem device easy to incorporate into their daily routines, unlike therapies requiring professional administration, invasive procedures, or injections, such as mesotherapy [24] and PRP [25].

Previous studies on the mechanisms of electrical stimulation to promote tissue regeneration and ETG have indicated different possible routes of action including increased secretion of vascular endothelial growth factor and keratinocyte growth factor [12], the formation of microvasculature and collagen in the scalp [14, 26], enhanced epidermal proliferation [27], increased blood flow, and increased trichogenic activity of dermal papilla cells [11]. Likewise, it has been suggested that HFSCs, which are the skin stem cells responsible for hair growth, are responsive to specific electrical currents, similar to the way soft tissue wounds and fractures heal [14, 16, 28, 29]. In vitro and ex vivo, HFSCs increase their numbers, proliferation, and activated hair regeneration genetic pathways upon electrical stimulation (Chacón‐Martínez, unpublished), further supporting the evidence of the important role of HFSCs in ETG. Given that the niostem device increased vellus hairs within 3 months and progressively caused an increase in total hair density, terminal hair density, and hair thickness over 6 months (Figures 2 and 4), we speculate that the device ultimately promotes HFSC activation, resulting in the entry of dormant hair follicles into the anagen phase. The results also suggest that the effects of the device could be cumulative over time. Future studies exploring how the niostem device enhances hair growth at the mechanistic level will help maximize its benefits.

In line with previous ETG studies [15, 16], we found no adverse side effects from prolonged use of the niostem device. Given its safe profile and the fact that > 25% of adult women experience AGA during their lifetime [3], testing the device in female AGA patients would be valuable. Additionally, since hair loss is a common side effect of chemotherapy [30], and minoxidil is almost the sole treatment for women undergoing chemotherapy [10], we suggest that the niostem device may benefit cancer patients suffering from hair loss, as ETG has been shown to reduce hair loss in female breast cancer patients [14].

Like many early‐stage pilot studies, the present work had limitations, including a small sample size (*n* = 21), the absence of a control group, a relatively narrow age range (20–40 years), and reliance on self‐reported adherence to the daily use of the device. These factors naturally limit generalizing the results at the population level, but they also guide future research. Despite the limitations, the study lays an important foundation for this novel approach to treating androgenetic alopecia.

## 5 Conclusion

In conclusion, this pilot study provides preliminary evidence that the niostem device is effective and safe for the treatment of androgenetic alopecia in men. The significant improvements in hair density, thickness, and terminal hair count observed over a 6‐month period suggest that low‐level electrical stimulation is a promising alternative approach to combat hair loss. While these results are very encouraging, further larger randomized controlled trials with more diverse participants and automated adherence monitoring will be needed to expand these findings at a larger scale and explore the long‐term efficacy and safety of the device.

## Conflicts of Interest

S.J. worked for and is a shareholder of Mane Biotech GmbH. S.M. declares no conflicts of interest. C.A.C.‐M. works for and is a shareholder of Mane Biotech GmbH. S.J. and C.A.C.‐M. are inventors on a patent for the electrotrichogenic device described in this study.

## Supporting information


**Table S1.** Changes in hair thickness and density over time in all participants.
**Figure S1.** Photographs of participants in the study.

## Data Availability

The data that supports the findings of this study are available in the Supporting Information of this article.
