# Supplementary material for: Novel Electrotrichogenic Device Promotes Hair Growth in Men With Androgenetic Alopecia: A Pilot Study
Source: J Cosmet Dermatol. 2025 Apr 28;24(5):e70202. doi: 10.1111/jocd.70202 (PMC12038312; doi:10.1111/jocd.70202)
Supplement: Supplementary file 1 — Table S1. Changes in hair thickness and density over time in all participants. Figure S1. Photographs of participants in the study. [file JOCD-24-e70202-s001.docx]

**Supplementary information**

**Figure S1.** Photographs of
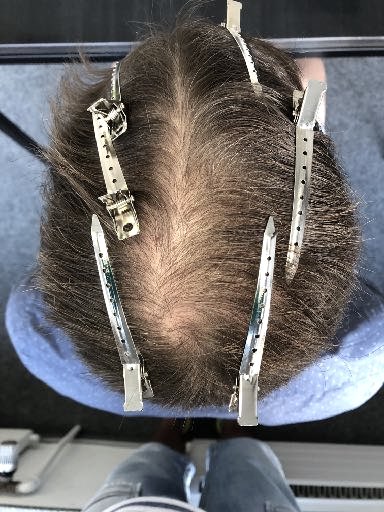

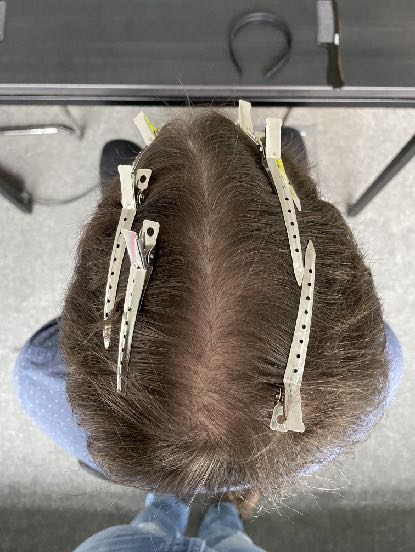

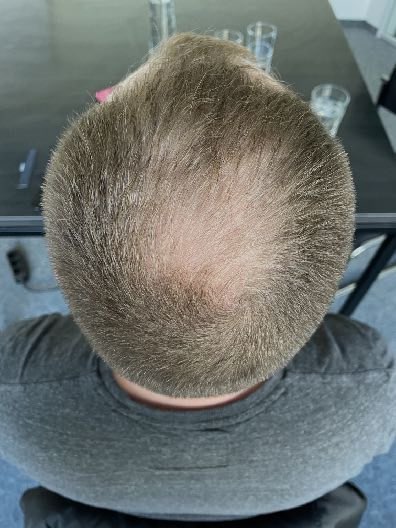

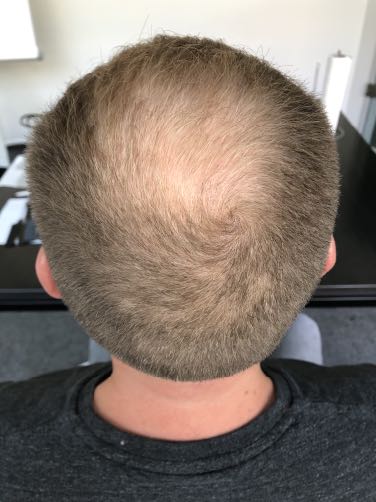

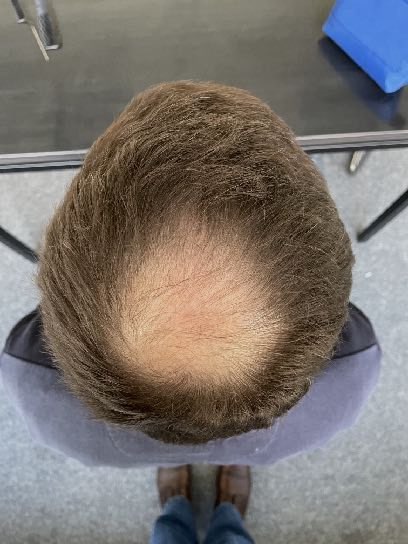

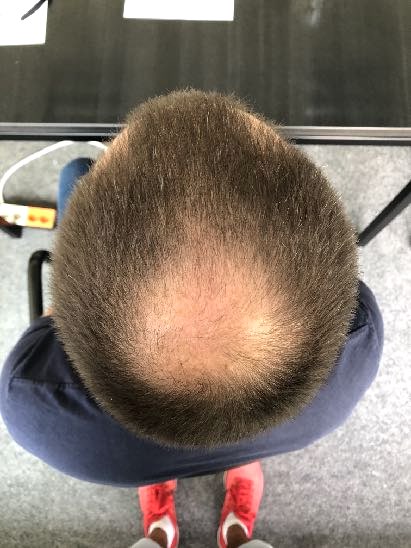
participants in the study

6 month

0 month

6 month

0 month


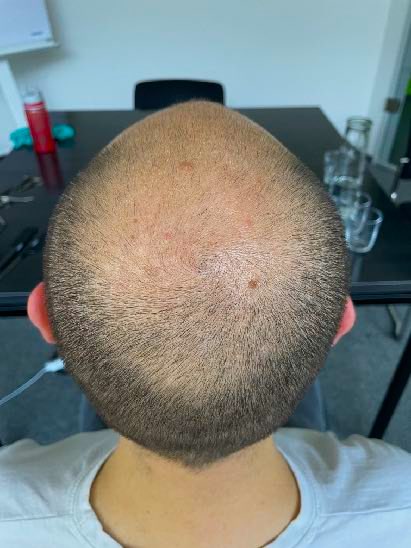

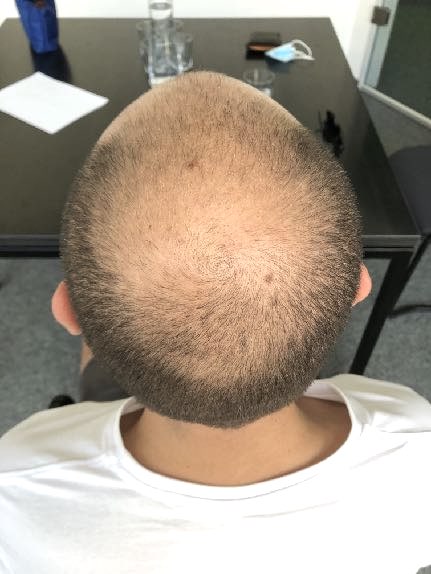

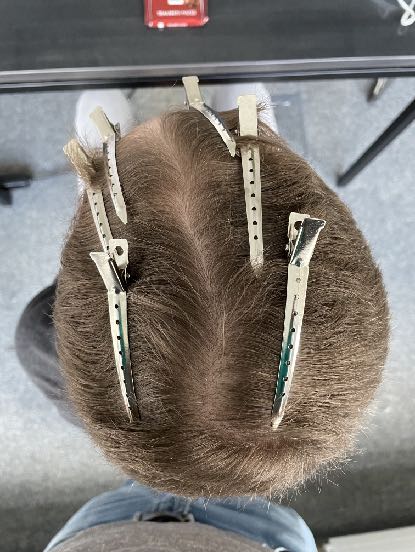

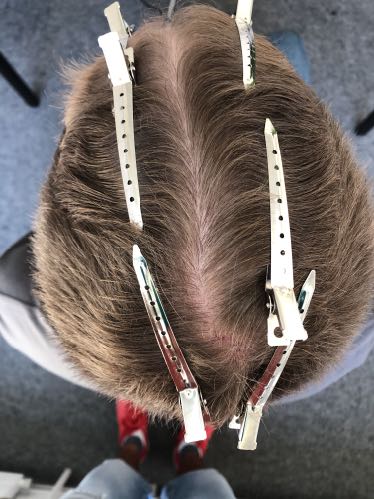

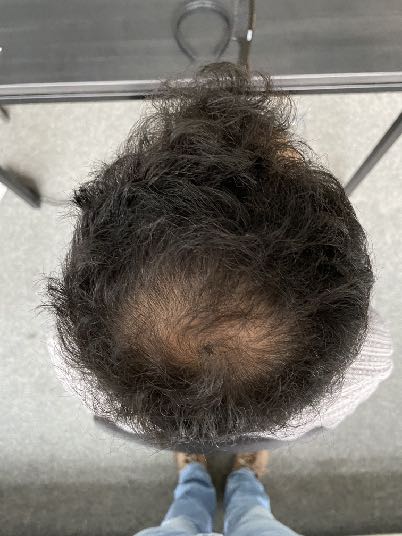

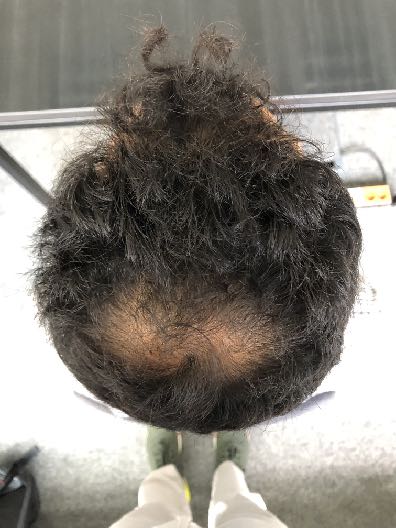


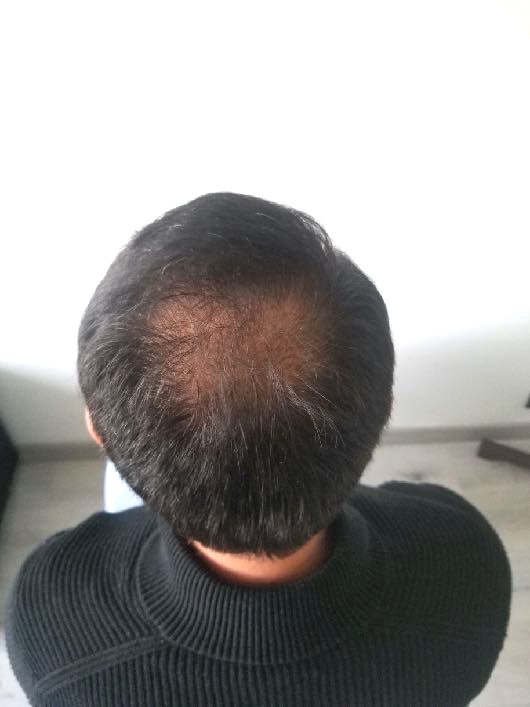

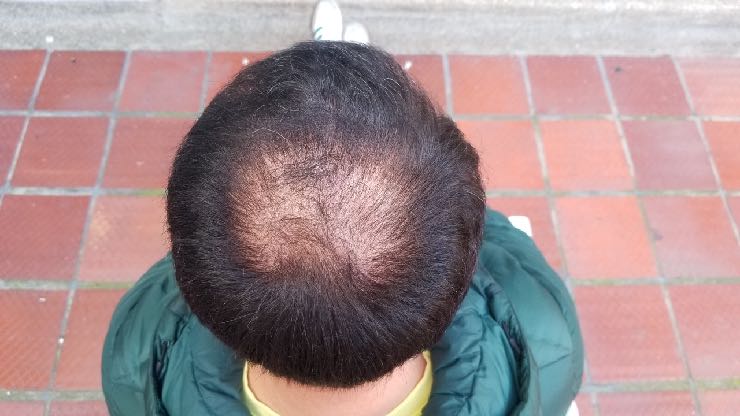

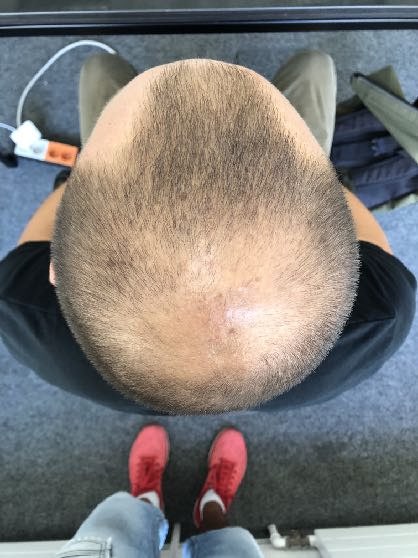

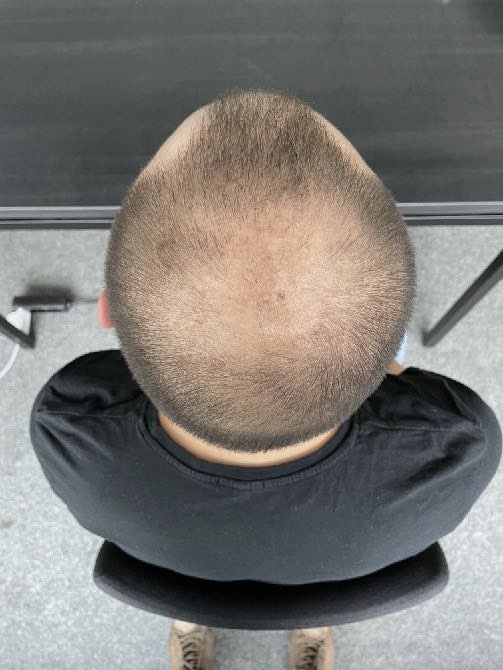

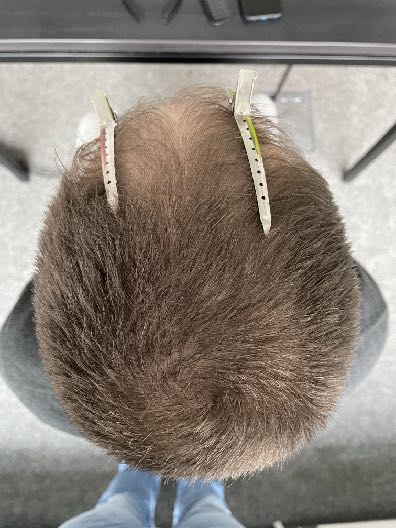

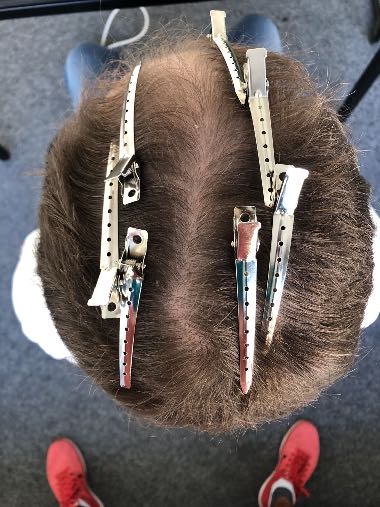


6 month

0 month

6 month

0 month


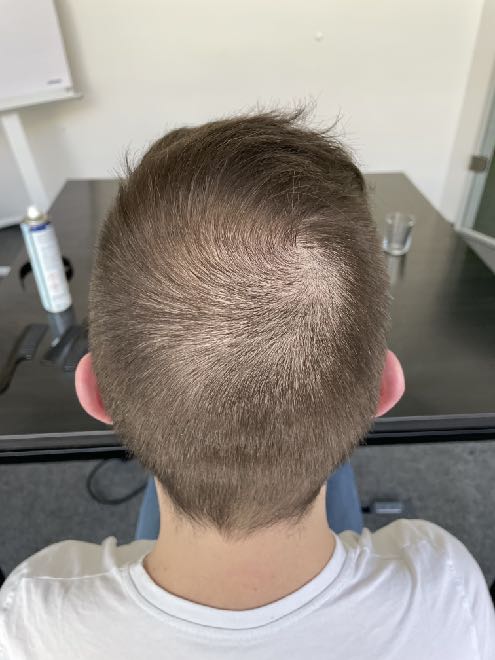

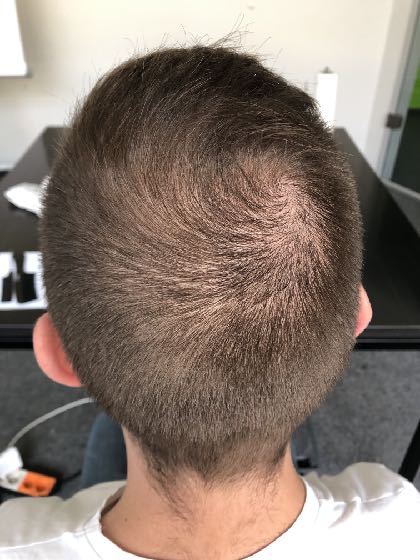

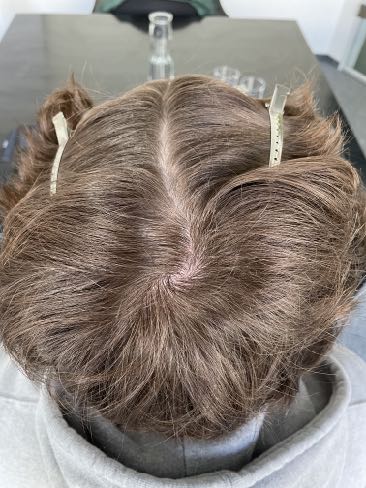

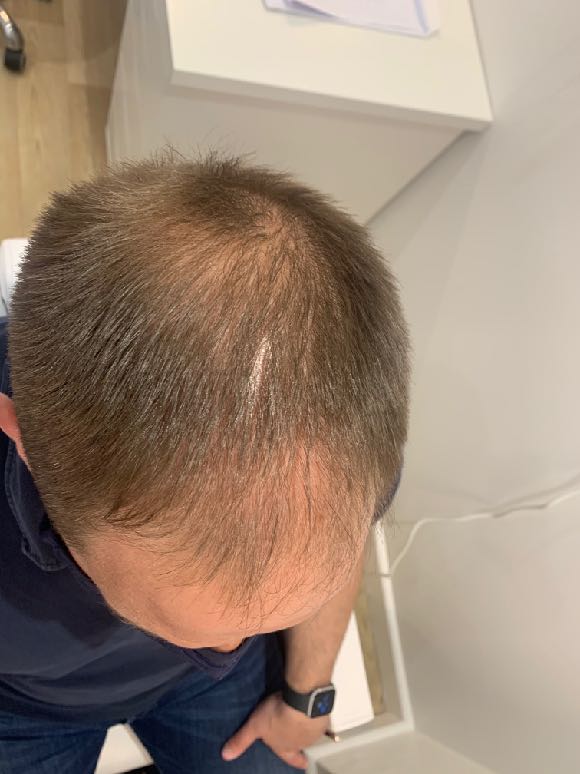

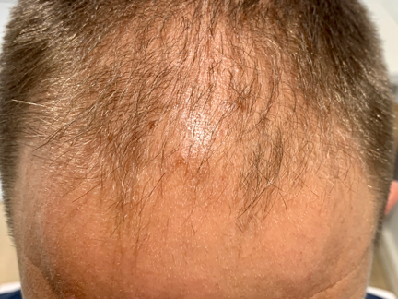

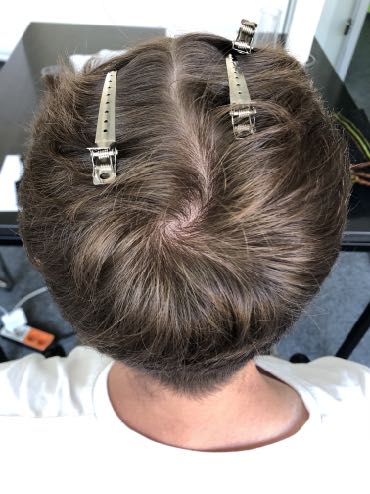


0 month

6 month


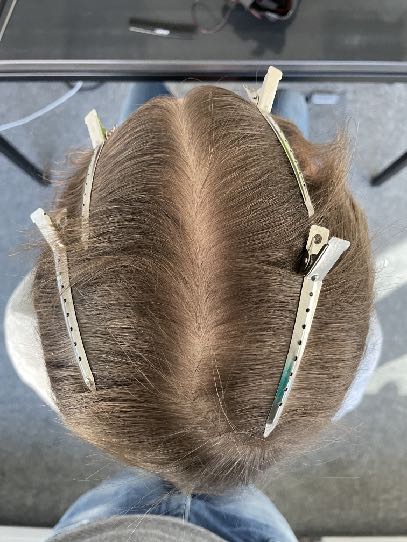

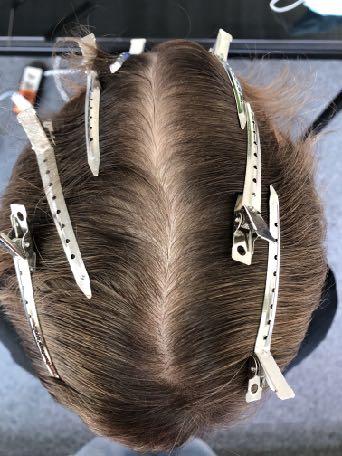

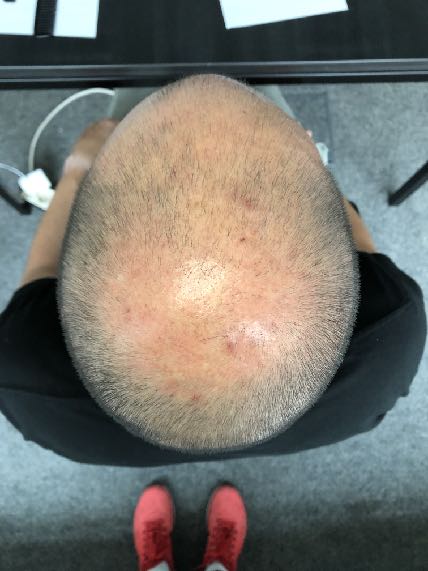

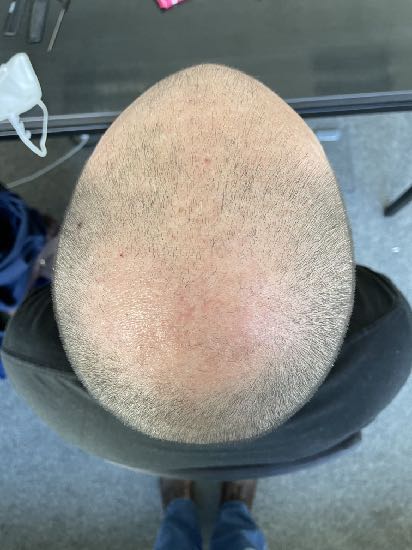

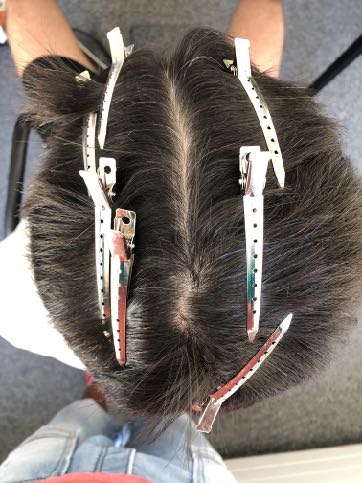

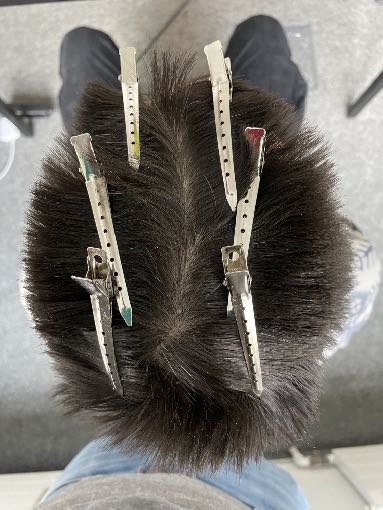


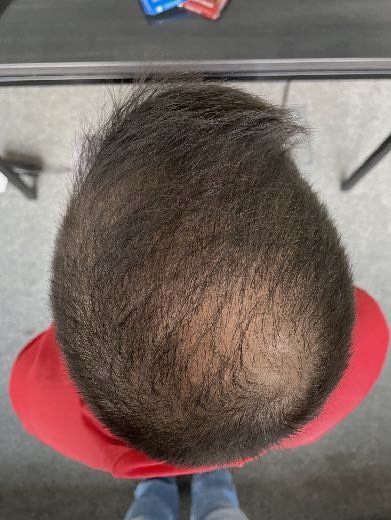

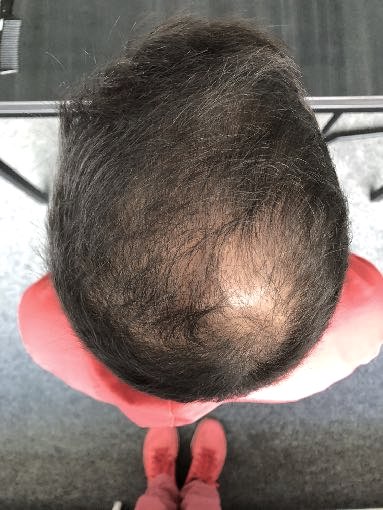

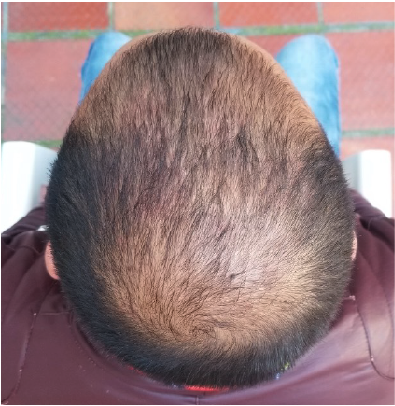

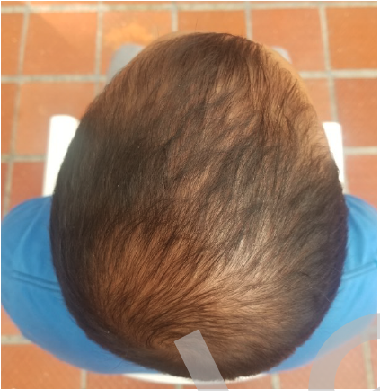

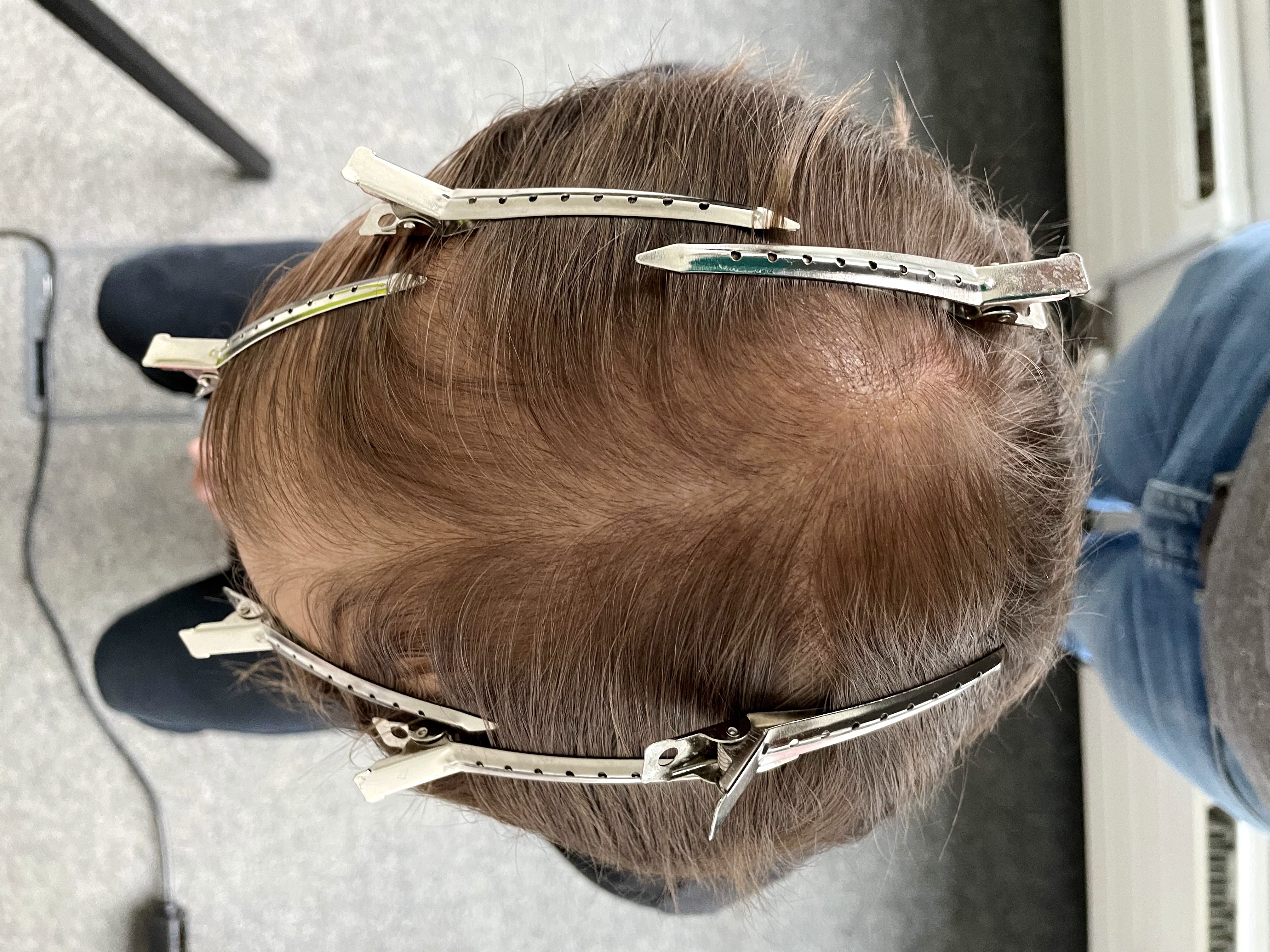

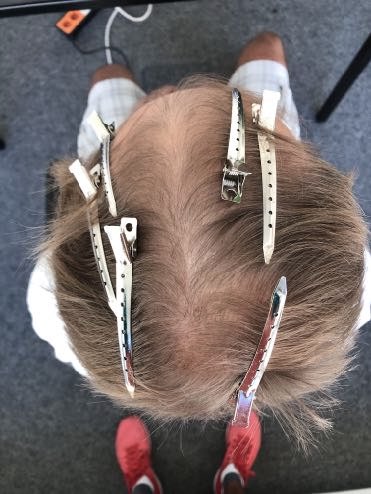


6 month

0 month


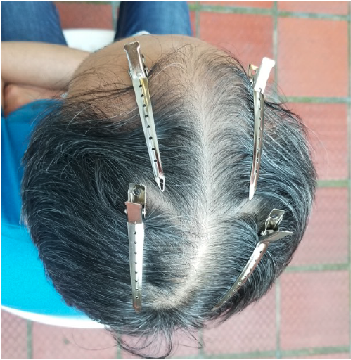

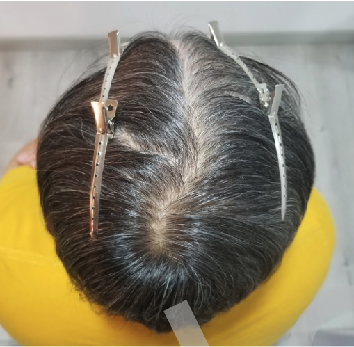


6 month

0 month


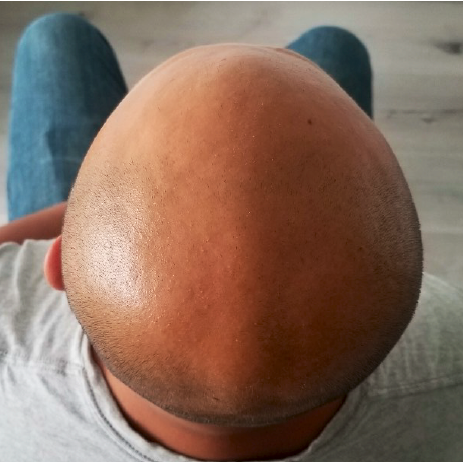

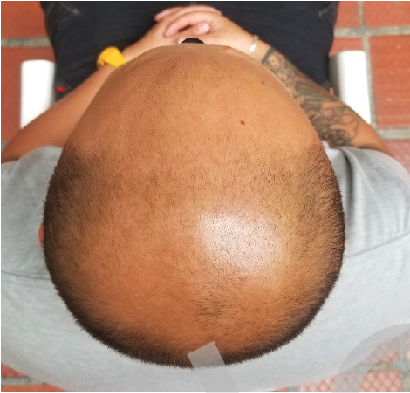


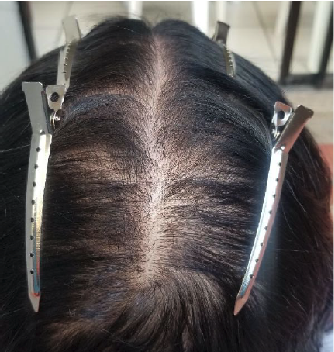

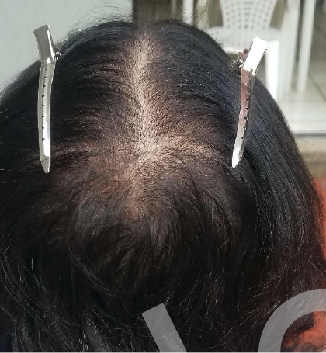


**Supplementary Table S1.** Changes in hair thickness and hair density over time. Hair density is shown for total, terminal and vellus hairs. Data from all study participants is shown.

|  | **Total hair density (hairs/cm^2^)** | | | **Terminal hair density (hairs/cm^2^)** | | | **Vellus hair density (hairs/cm^2^)** | | | **Cumulative thickness (µm)** | | |
| --- | --- | --- | --- | --- | --- | --- | --- | --- | --- | --- | --- | --- |
| **Subject** | **0 month** | **3 months** | **6 months** | **0 month** | **3 months** | **6 months** | **0 month** | **3 months** | **6 months** | **0 month** | **3 months** | **6 months** |
| 1 | 275 | 323 | 346 | 76 | 57 | 69 | 199 | 266 | 278 | 8919,8 | 8100,6 | 8575,5 |
| 2 | 281 | 369 | 350 | 67 | 58 | 71 | 214 | 312 | 279 | 7734,7 | 7663,2 | 8818,9 |
| 3 | 209 | 220 | 231 | 176 | 140 | 174 | 33 | 79 | 57 | 10644,3 | 9444,6 | 10878,8 |
| 4 | 155 | 155 | 176 | 34 | 28 | 35 | 121 | 127 | 141 | 4156,6 | 3337,7 | 3833,8 |
| 5 | 192 | 248 | 225 | 24 | 15 | 21 | 168 | 233 | 204 | 4036,1 | 3986,2 | 3956,8 |
| 6 | 187 | 202 | 231 | 45 | 47 | 53 | 142 | 155 | 177 | 5230,3 | 5384,5 | 5692,1 |
| 7 | 270 | 277 | 332 | 216 | 192 | 262 | 57 | 76 | 64 | 13585,3 | 12729,3 | 17419,5 |
| 8 | 178 | 192 | 255 | 103 | 94 | 111 | 74 | 98 | 145 | 8156,6 | 8256,6 | 9577,0 |
| 9 | 344 | 371 | 402 | 86 | 82 | 80 | 258 | 289 | 322 | 10727,7 | 11163,5 | 11747,3 |
| 10 | 221 | 257 | 263 | 54 | 55 | 60 | 166 | 202 | 204 | 6398,2 | 6617,9 | 6819,9 |
| 11 | 311 | 352 | 368 | 153 | 140 | 160 | 157 | 211 | 208 | 11584,7 | 11987,6 | 13035,2 |
| 12 | 221 | 229 | 258 | 129 | 122 | 139 | 92 | 107 | 119 | 8944,0 | 8454,7 | 9180,3 |
| 13 | 203 | 273 | 289 | 55 | 49 | 70 | 148 | 224 | 219 | 6152,9 | 6974,1 | 7761,5 |
| 14 | 178 | 197 | 191 | 22 | 20 | 16 | 156 | 177 | 175 | 4575,1 | 4284,1 | 3870,3 |
| 15 | 158 | 173 | 181 | 23 | 27 | 37 | 124 | 146 | 144 | 3073,5 | 3355,6 | 3944,8 |
| 16 | 258 | 261 | 281 | 104 | 147 | 194 | 155 | 114 | 85 | 8906,2 | 10561,2 | 13586,3 |
| 18 | 133 | 159 | 153 | 75 | 33 | 24 | 60 | 126 | 129 | 5590,9 | 4349,2 | 4056,2 |
| 19 | 155 | 184 | 170 | 83 | 76 | 82 | 72 | 106 | 88 | 7648,9 | 6652,9 | 6533,1 |
| 20 | 163 | 196 | 207 | 128 | 141 | 157 | 36 | 55 | 50 | 9706,5 | 10217,6 | 11543,3 |
| 21 | 85 | 93 | dropout | 28 | 13 | dropout | 58 | 80 | dropout | 2603,7 | 2221,3 | dropout |
| 22 | 266 | 220 | 289 | 109 | 101 | 152 | 157 | 119 | 136 | 9213,3 | 8006,1 | 10997,3 |
